# Supplementary material for: Molecular Docking and Biophysical Studies for Antiproliferative Assessment of Synthetic Pyrazolo-Pyrimidinones Tethered with Hydrazide-Hydrazones
Source: Int J Mol Sci. 2021 Mar 8;22(5):2742. doi: 10.3390/ijms22052742 (PMC7962976; doi:10.3390/ijms22052742)
Supplement: Supplementary file 1 [file ijms-22-02742-s001.pdf]

International Journal of
Molecular Sciences

MDPI

Supplementary material

Molecular docking and biophysical studies for antiproliferative assessment of synthetic pyrazolo-pyrimidinones tethered with hydrazide-hydrazones

Table of Contents

Figure S1. Real time monitoring of NHDF cell viability .... p. 2
Figure S2. Binding mode of ester **3** in the erlotinib binding site of EGFR (PDB:1M17) .... p. 2
Figure S3. CD spectra of a) KRAS 22RT, b) BCL2-G4, c) Tel 23 and d) Hairpin duplex. .... p. 3
Figure S4. ^{1}H NMR spectrum of compound **2** (DMSO-d_6, 400MHz) .... p. 4
Figure S5. ^{13}C NMR spectrum of compound **2** (DMSO-d_6, 100MHz) .... p. 4
Figure S6. ^{1}H NMR spectrum of compound **3** (DMSO-d_6, 400MHz) .... p. 5
Figure S7. ^{13}C NMR spectrum of compound **3** (DMSO-d_6, 100MHz) .... p. 5
Figure S8. ^{1}H NMR spectrum of compound **4** (DMSO-d_6, 400MHz) .... p. 6
Figure S9. ^{13}C NMR spectrum of compound **4** (DMSO-d_6, 100MHz) .... p. 6
Figure S10. ^{1}H NMR spectrum of compound **5a** (DMSO-d_6, 400MHz) .... p. 7
Figure S11. ^{13}C NMR spectrum of compound **5a** (DMSO-d_6, 100MHz) .... p. 7
Figure S12. ^{1}H NMR spectrum of compound **5b** (DMSO-d_6, 400MHz) .... p. 8
Figure S13. ^{13}C NMR spectrum of compound **5b** (DMSO-d_6, 100MHz) .... p. 8
Figure S14. ^{1}H NMR spectrum of compound **5c** (DMSO-d_6, 400MHz). .... p. 9
Figure S15. ^{13}C NMR spectrum of compound **5c** (DMSO-d_6, 100MHz) .... p. 9
Figure S16. ^{1}H NMR spectrum of compound **5d** (DMSO-d_6, 400MHz) .... p. 10
Figure S17. ^{13}C NMR spectrum of compound **5d** (DMSO-d_6, 100MHz) .... p. 10
Figure S18. ^{1}H NMR spectrum of compound **5e** (DMSO-d_6, 400MHz) .... p. 11
Figure S19. ^{13}C NMR spectrum of compound **5e** (DMSO-d_6, 100MHz) .... p. 11
Figure S20. ^{1}H NMR spectrum of compound **5f** (DMSO-d_6, 400MHz) .... p. 12
Figure S21. ^{13}C NMR spectrum of compound **5f** (DMSO-d_6, 100MHz) .... p. 12
Figure S22. ^{1}H NMR spectrum of compound **5g** (DMSO-d_6, 400MHz) .... p. 13
Figure S23. ^{13}C NMR spectrum of compound **5g** (DMSO-d_6, 100MHz) .... p. 13
Figure S24. ^{1}H NMR spectrum of compound **5h** (DMSO-d_6, 400MHz) .... p. 14
Figure S25. ^{13}C NMR spectrum of compound **5h** (DMSO-d_6, 100MHz) .... p. 14
Figure S26. HR-ESI-MS spectrum of compound **2**. .... p. 15
Figure S27. HR-ESI-MS spectrum of compound **3**. .... p. 15
Figure S28. HR-ESI-MS spectrum of compound **4**. .... p. 15
Figure S29. HR-ESI-MS spectrum of compound **5a**. .... p. 16
Figure S30. HR-ESI-MS spectrum of compound **5b**. .... p. 16
Figure S31. HR-ESI-MS spectrum of compound **5c**. .... p. 16
Figure S32. HR-ESI-MS spectrum of compound **5d**. .... p. 17
Figure S33. HR-ESI-MS spectrum of compound **5e**. .... p. 17
Figure S34. HR-ESI-MS spectrum of compound **5f** p.17
Figure S35. HR-ESI-MS spectrum of compound **5g** .... p. 18
Figure S36. HR-ESI-MS spectrum of compound **5h** .... p. 18

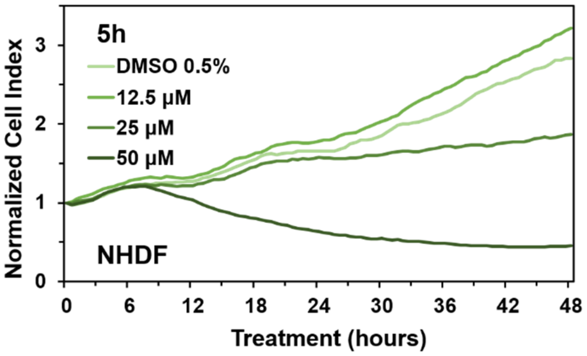

IC$_{50}$ - NHDF

Compound	IC$_{50}$ (µM) ± SE
5a	60.18 ± 2.908
5e	66.16 ± 3.367
5g	26.05 ± 1.110
5h	32.07 ± 1.703
CDDP	14.86 ± 0.802

Figure S1. Real time monitoring of NHDF cell viability after 48 h exposure to synthetic compounds **5a**, **5e**, **5g**, and **5h**, using the xCELLigence System Real-Time Cell Analyzer. Left panel, representative NCI traces of NHDF cells exposed to increasing concentrations (12.5, 25, 50 μM) of **5h** and 0.5% DMSO vehicle (control) for 48 hours. Right panel, IC$_{50}$ values (± standard error, SE) of cisplatin (CDDP), **5a**, **5e**, **5g**, and **5h** against NHDF cells after 48h drug exposure. The IC$_{50}$ value represents the concentration of each compound that reduces the NCI by 50%. Data are means of three independent experiments.

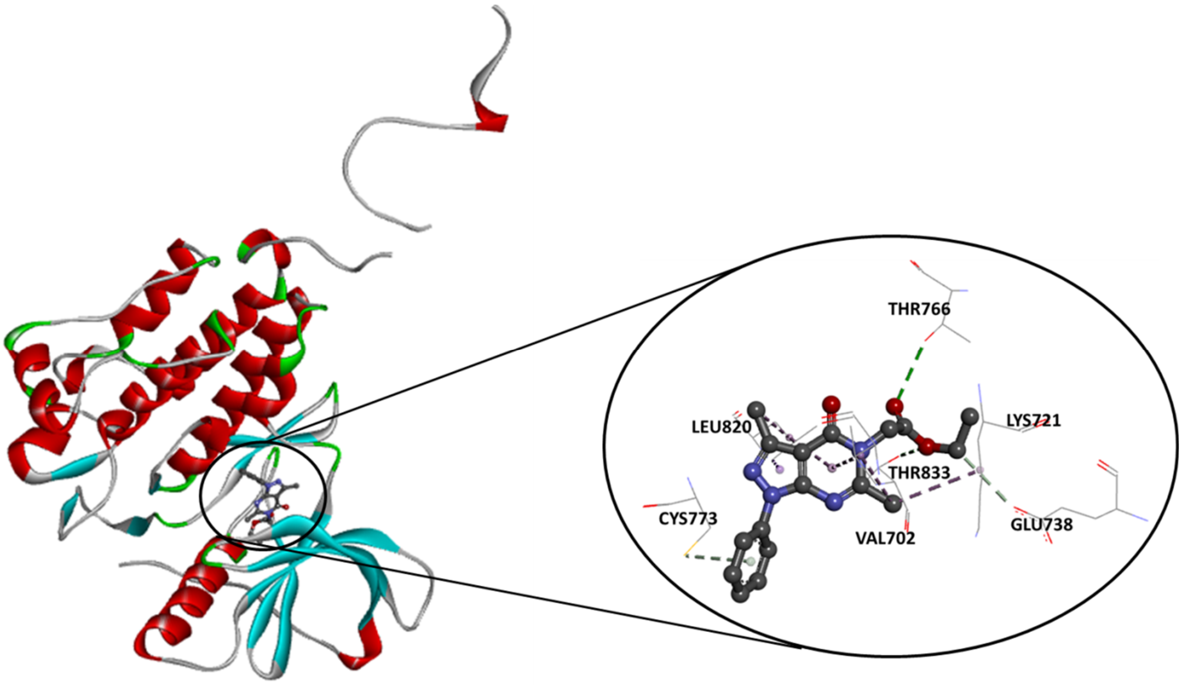

Figure S2. Binding mode of ester **3** in the Erlotinib binding site of EGFR (PDB:1M17).

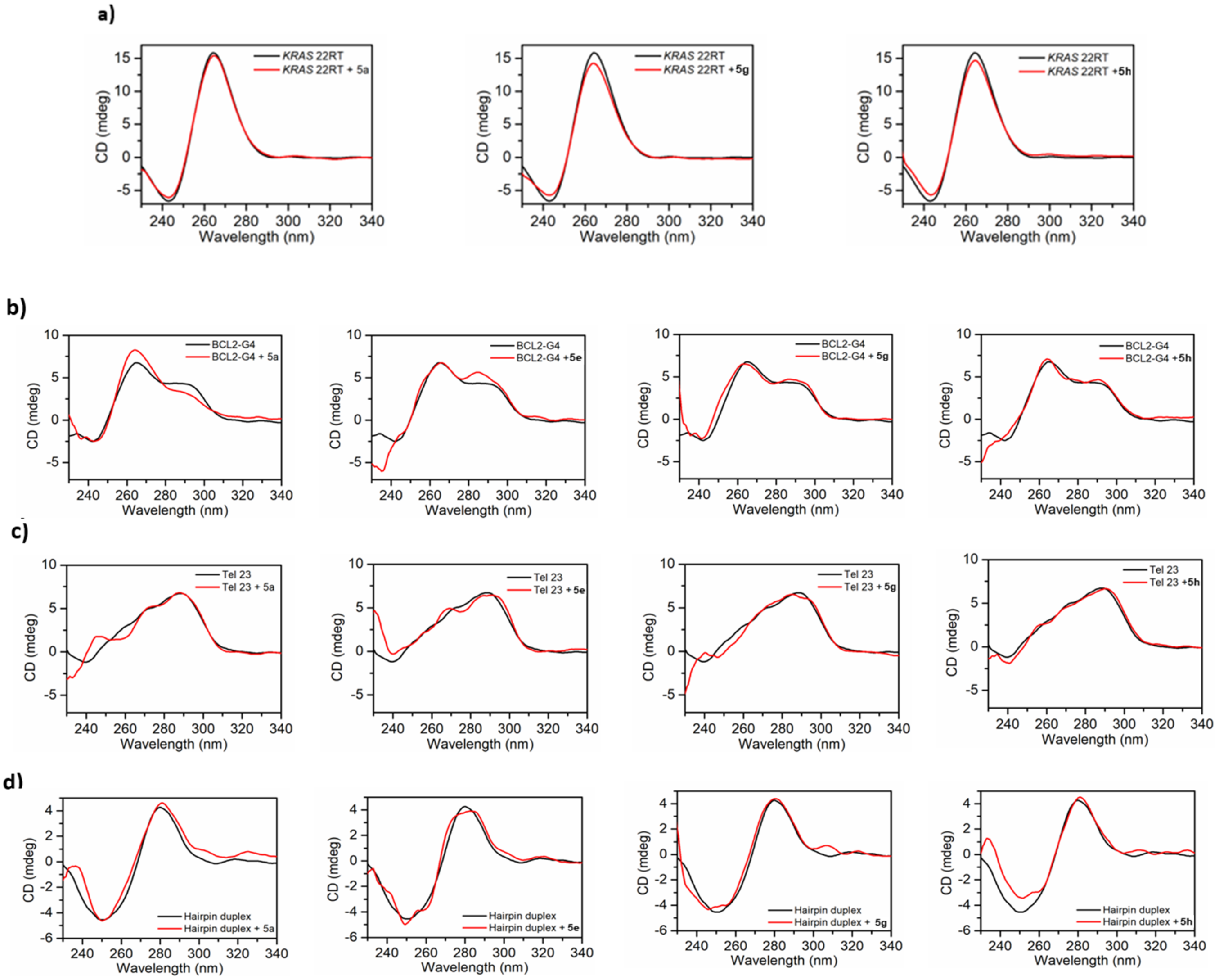

Figure S3. CD spectra of **a)** *KRAS* 22RT, **b)** BCL2-G4, **c)** Tel 23 and **d)** Hairpin duplex in the absence (black line) and in the presence (red line) of **5a**, **5e**, **5g** and **5h**.

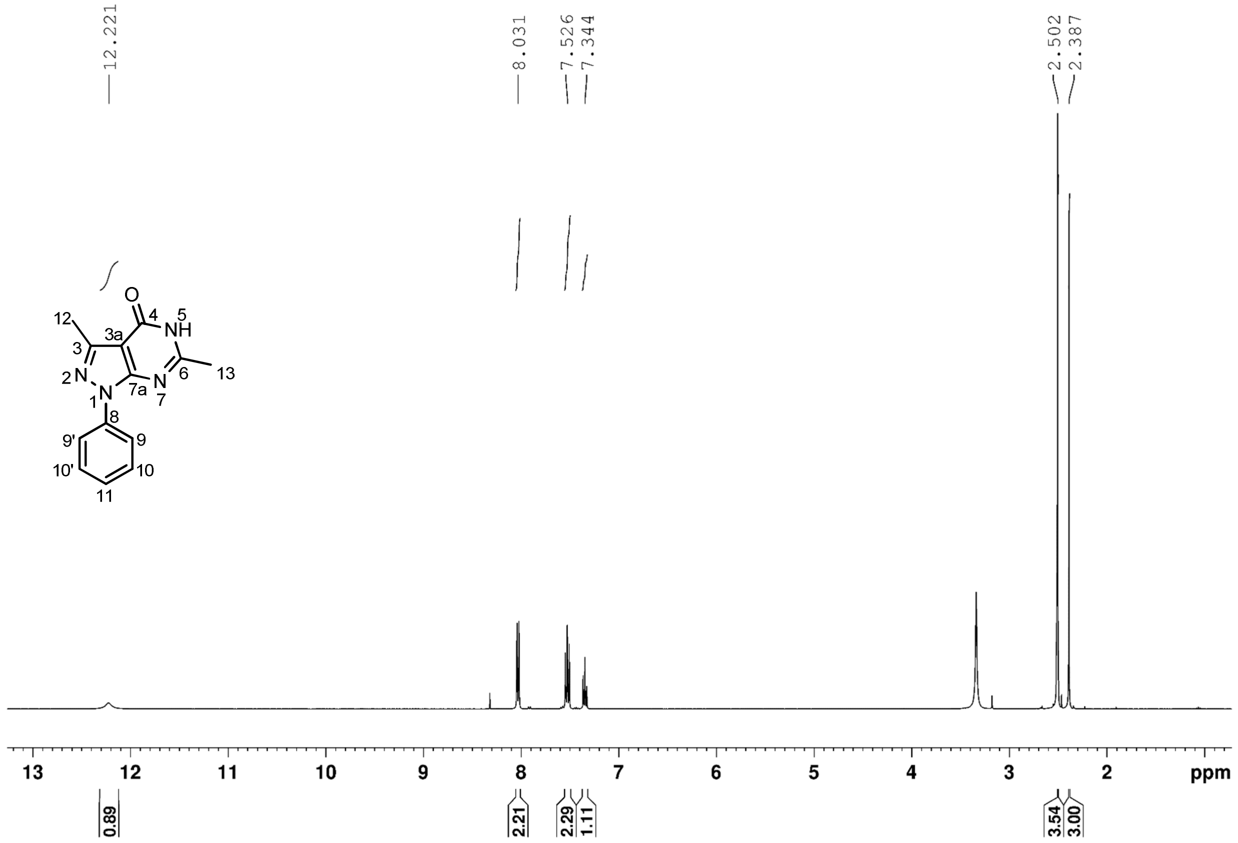

Figure S4. ^{1}H NMR spectrum of compound **2** (DMSO-*d*$_6$, 400MHz).

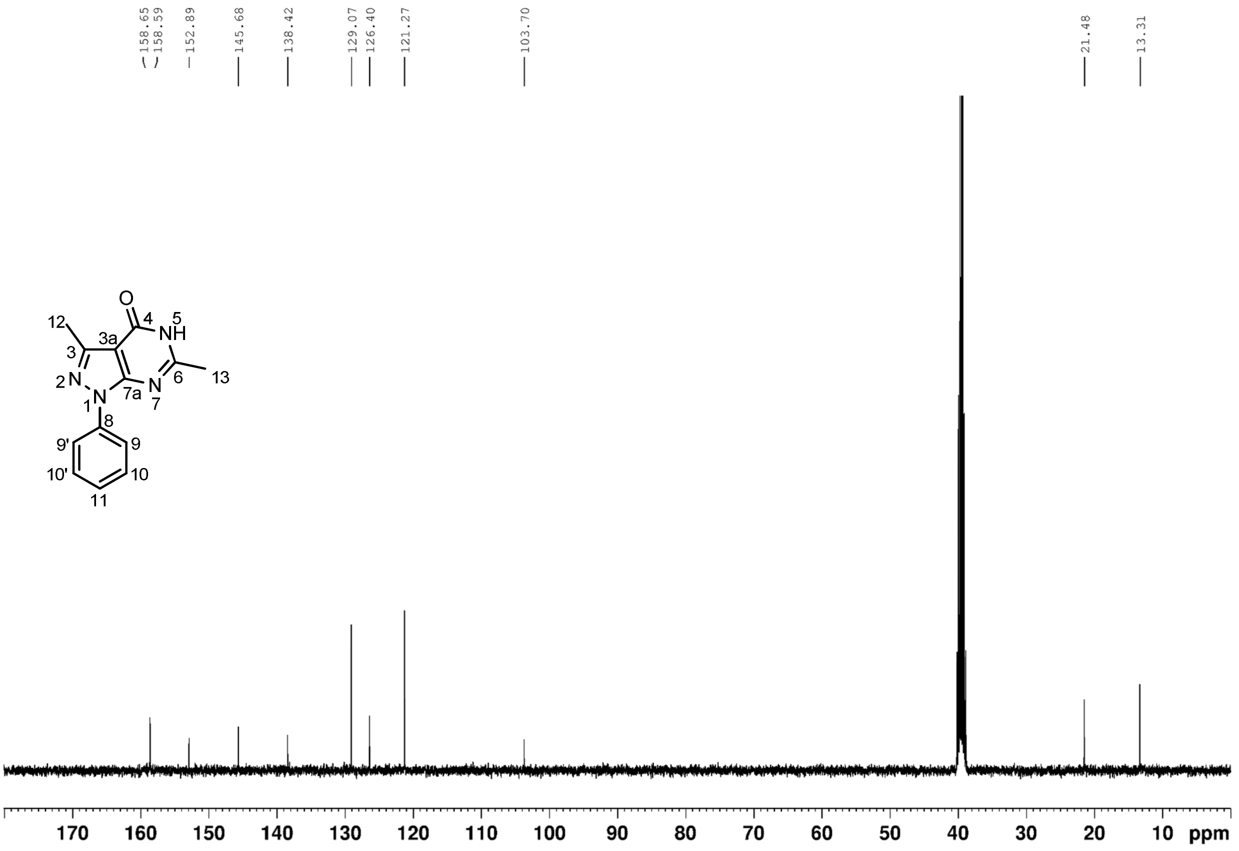

Figure S5. ^{13}C NMR spectrum of compound **2** (DMSO-*d*$_6$, 100MHz).

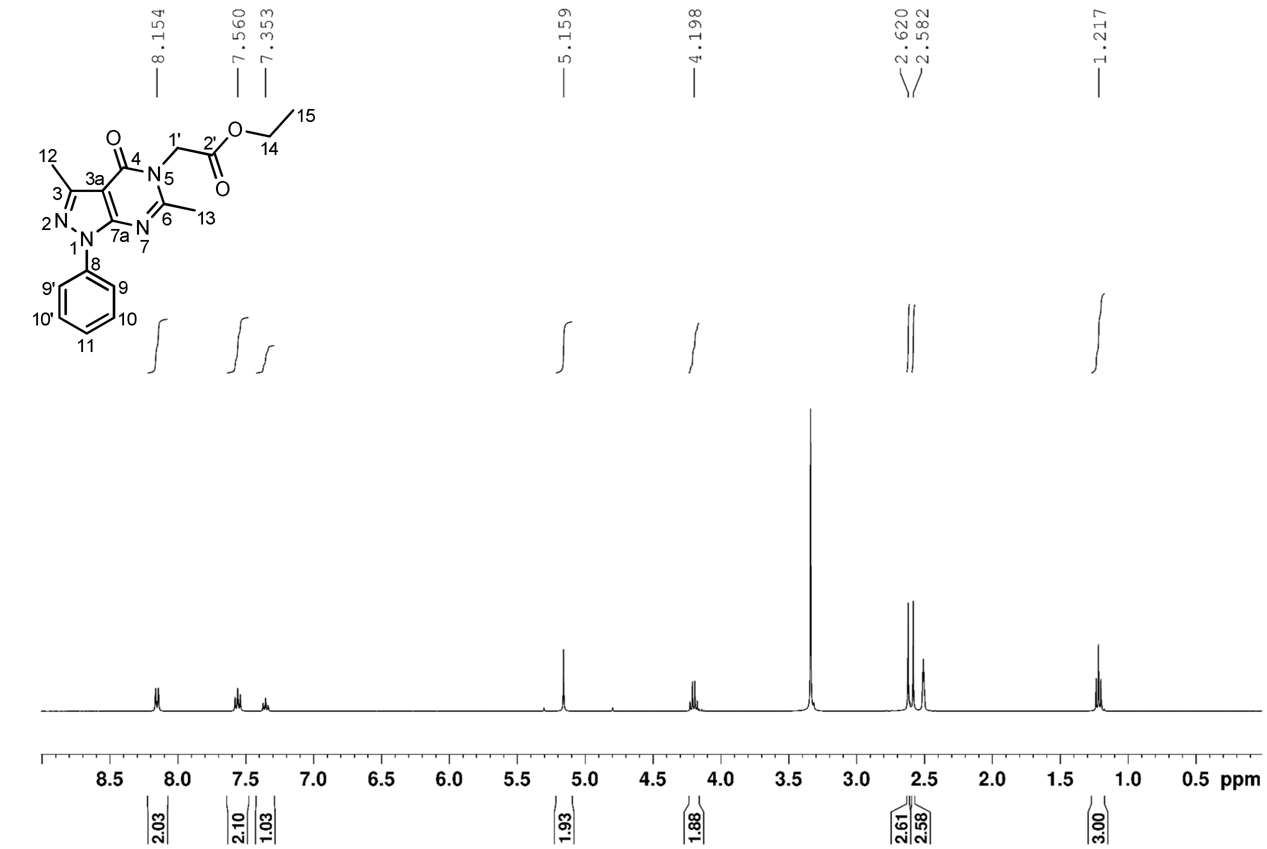

Figure S6. ^{1}H NMR spectrum of compound **3** (DMSO-d_6, 400MHz).

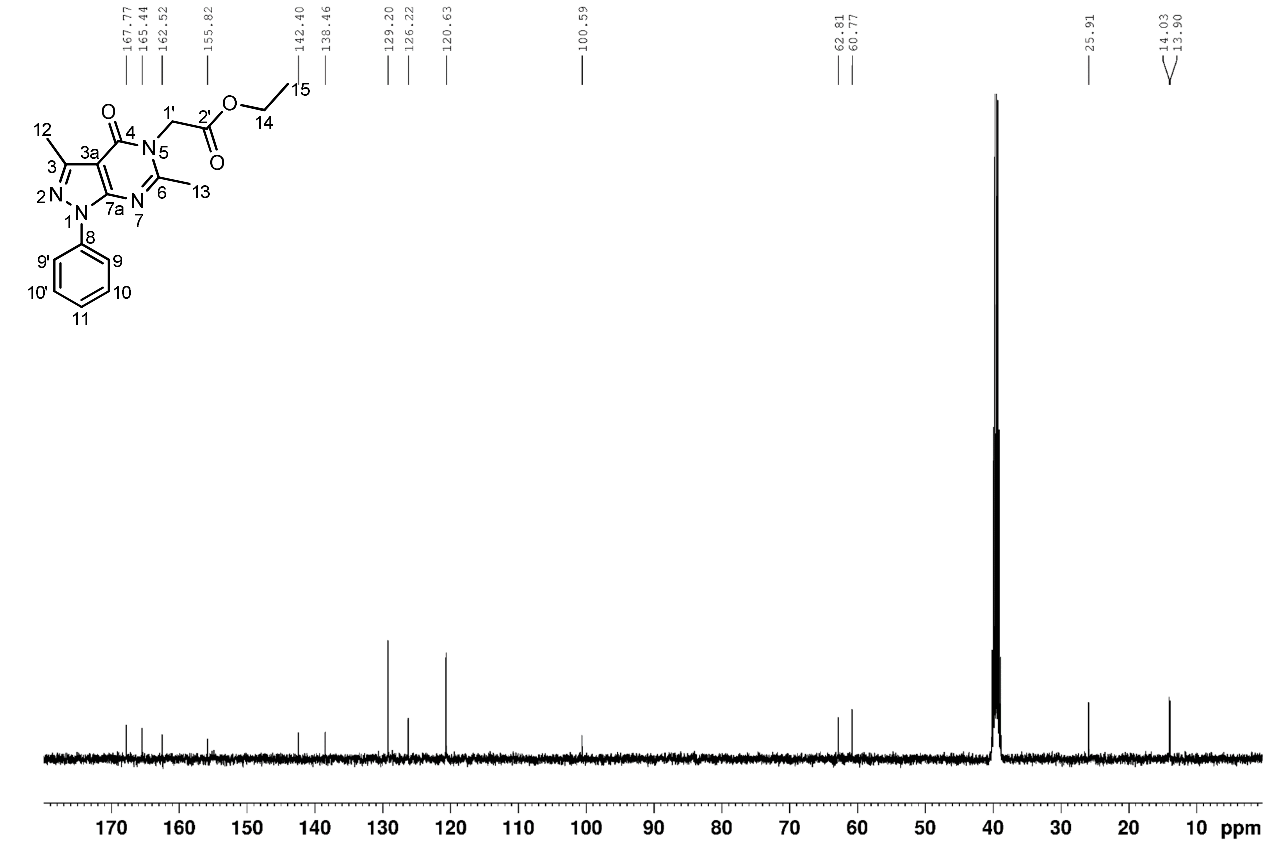

Figure S7. ^{13}C NMR spectrum of compound **3** (DMSO-d_6, 100MHz).

International Journal of
Molecular Sciences

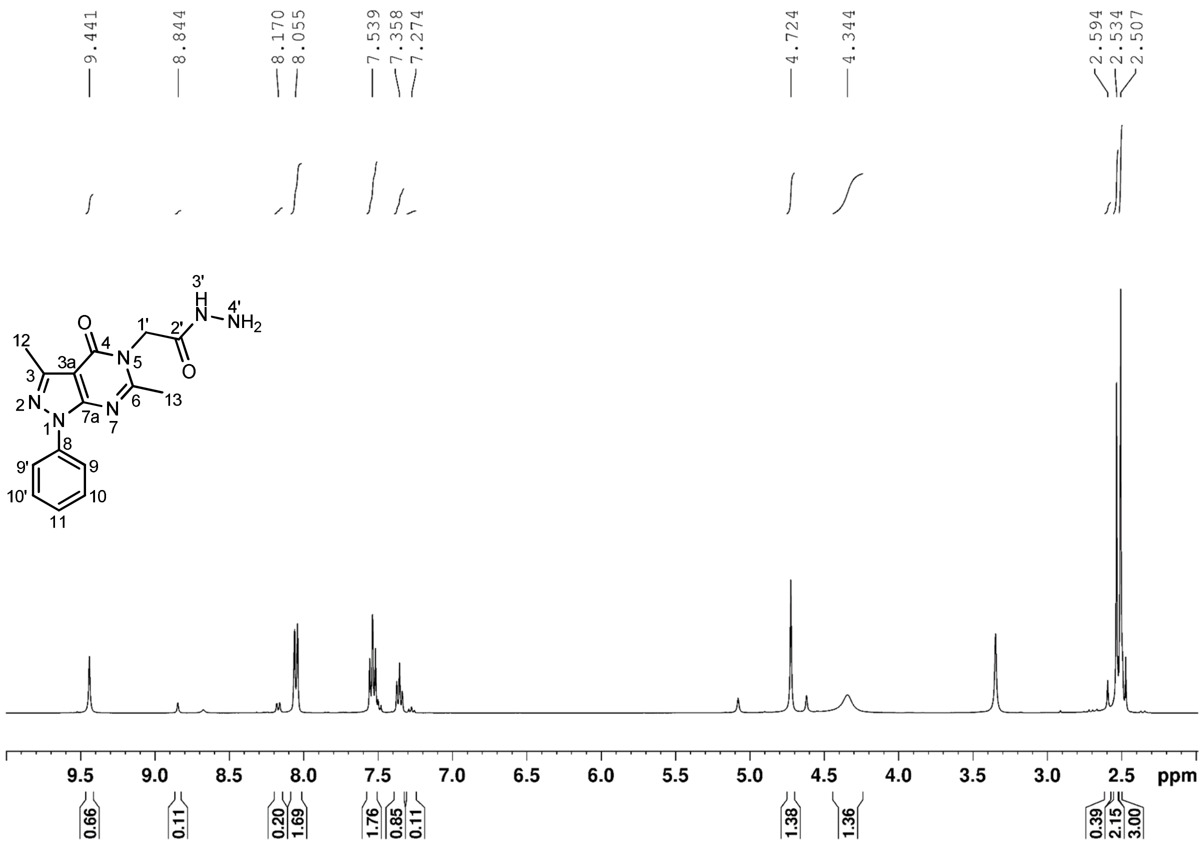

Figure S8. ^{1}H NMR spectrum of compound **4** (DMSO-d_6, 400MHz).

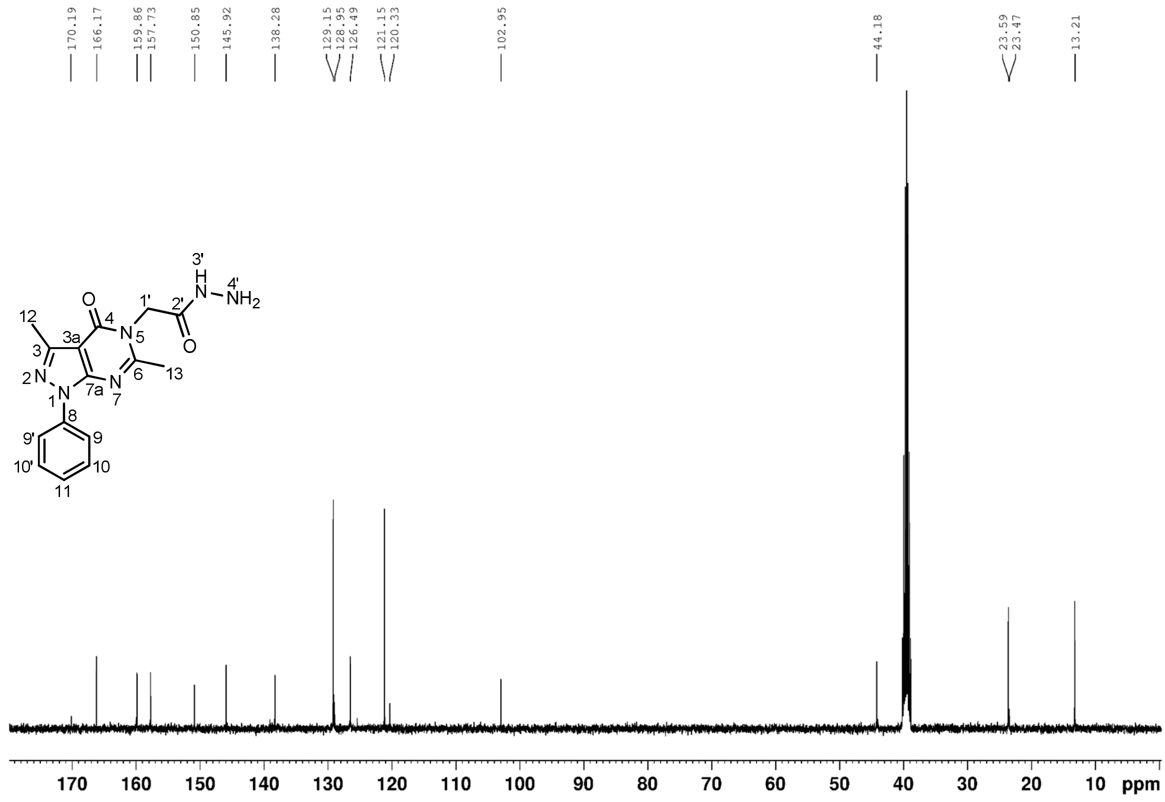

Figure S9. ^{13}C NMR spectrum of compound **4** (DMSO-d_6, 100MHz).

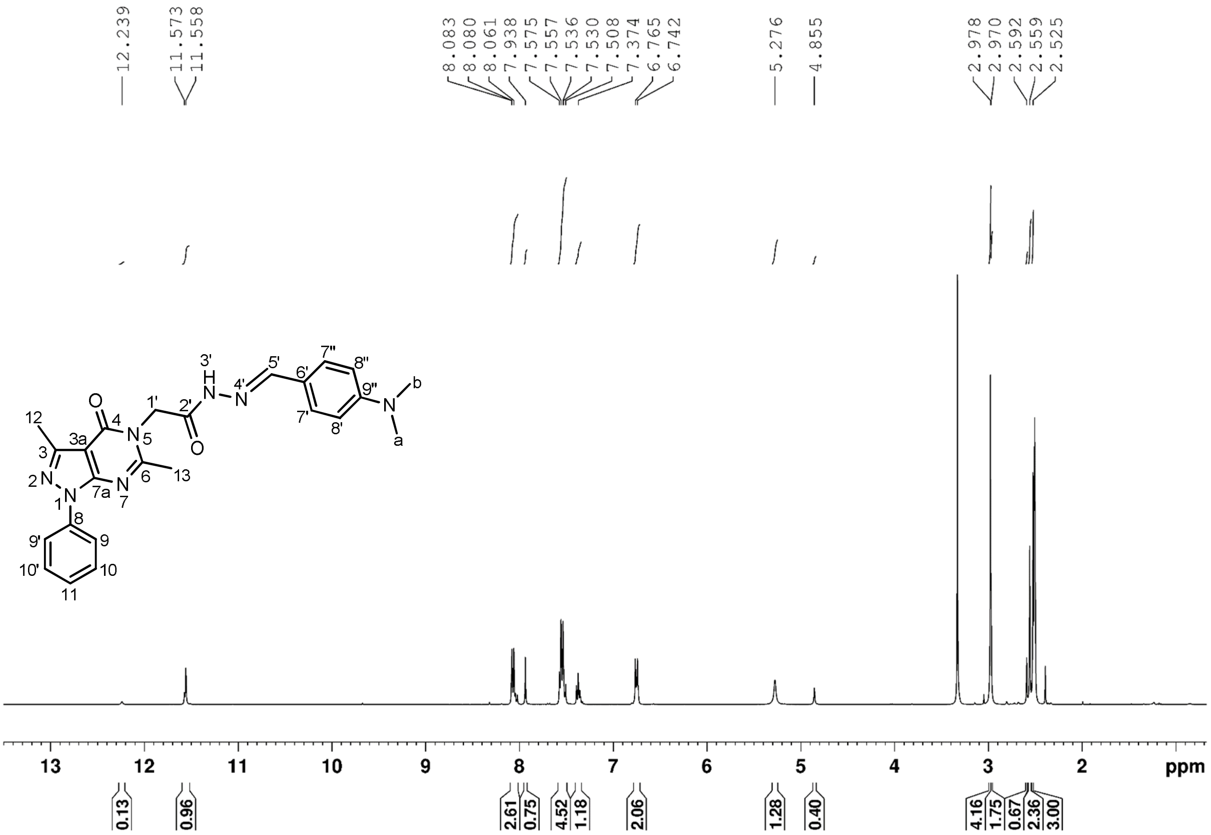

Figure S10. ^{1}H NMR spectrum of compound **5a** (DMSO-d_6, 400MHz).

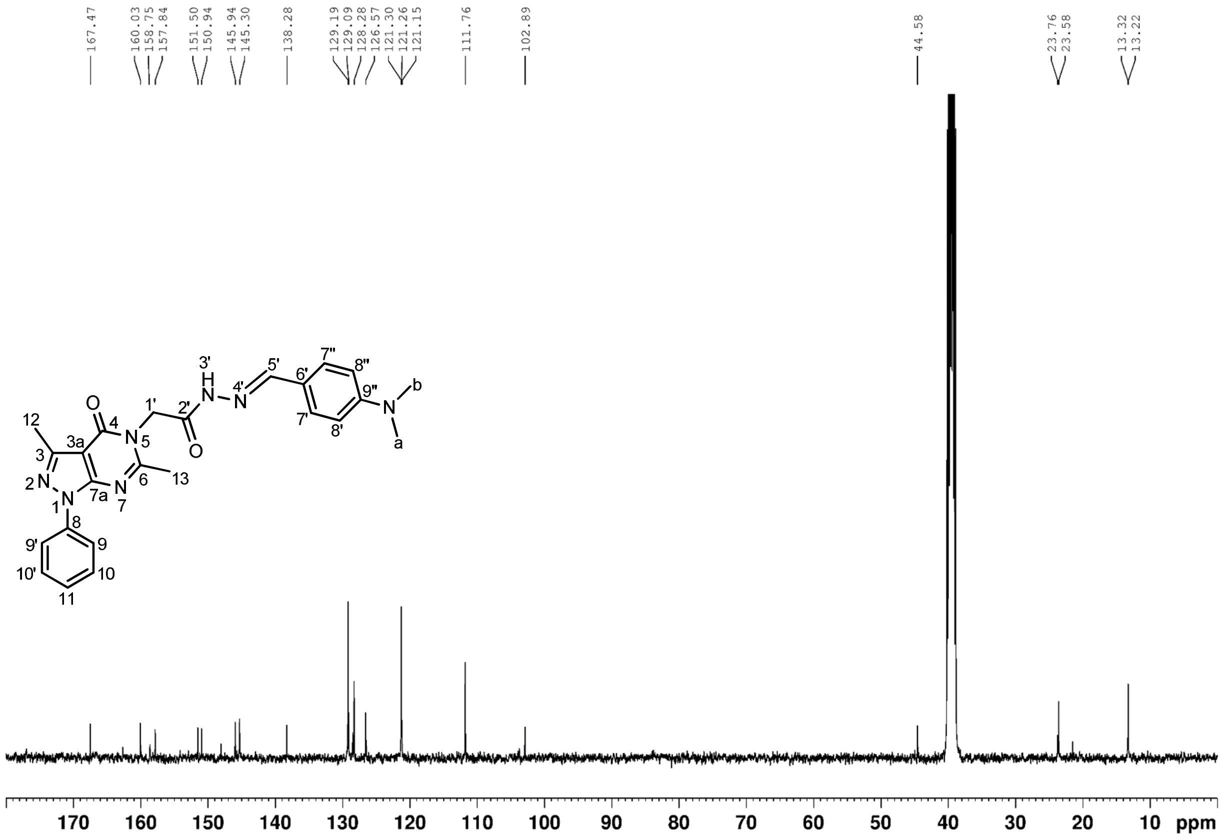

Figure S11. ^{13}C NMR spectrum of compound **5a** (DMSO-d_6, 100MHz).

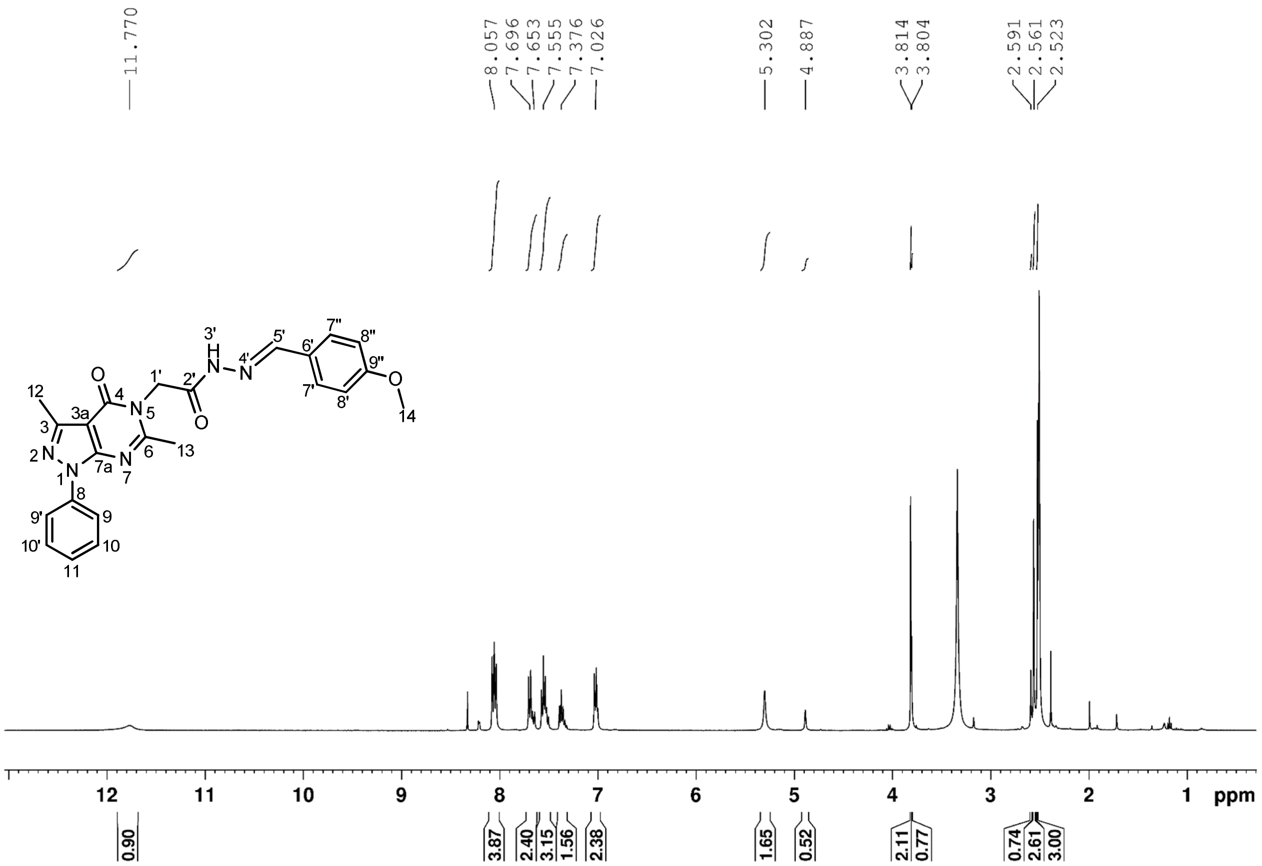

Figure S12. ^{1}H NMR spectrum of compound **5b** (DMSO-d_6, 400MHz).

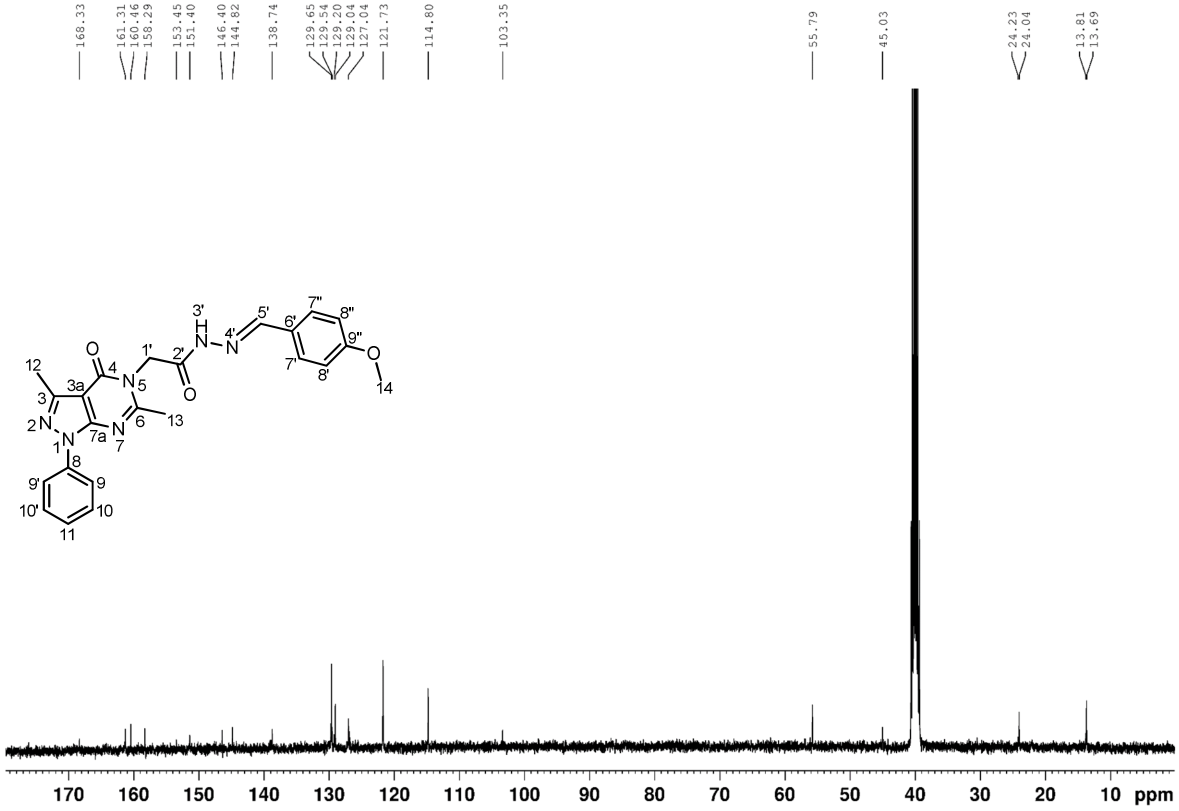

Figure S13. ^{13}C NMR spectrum of compound **5b** (DMSO-d_6, 100MHz).

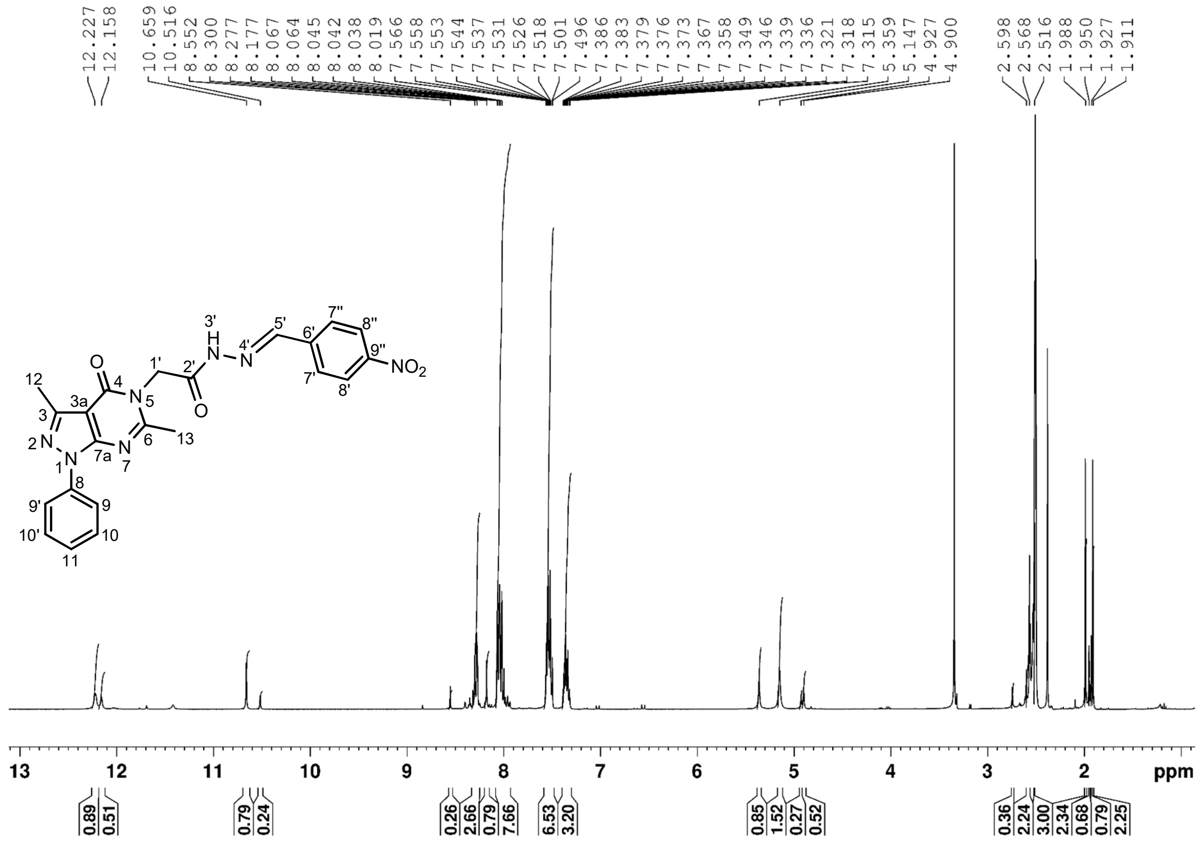

Figure S14. ^{1}H NMR spectrum of compound **5c** (DMSO-*d*$_6$, 400MHz).

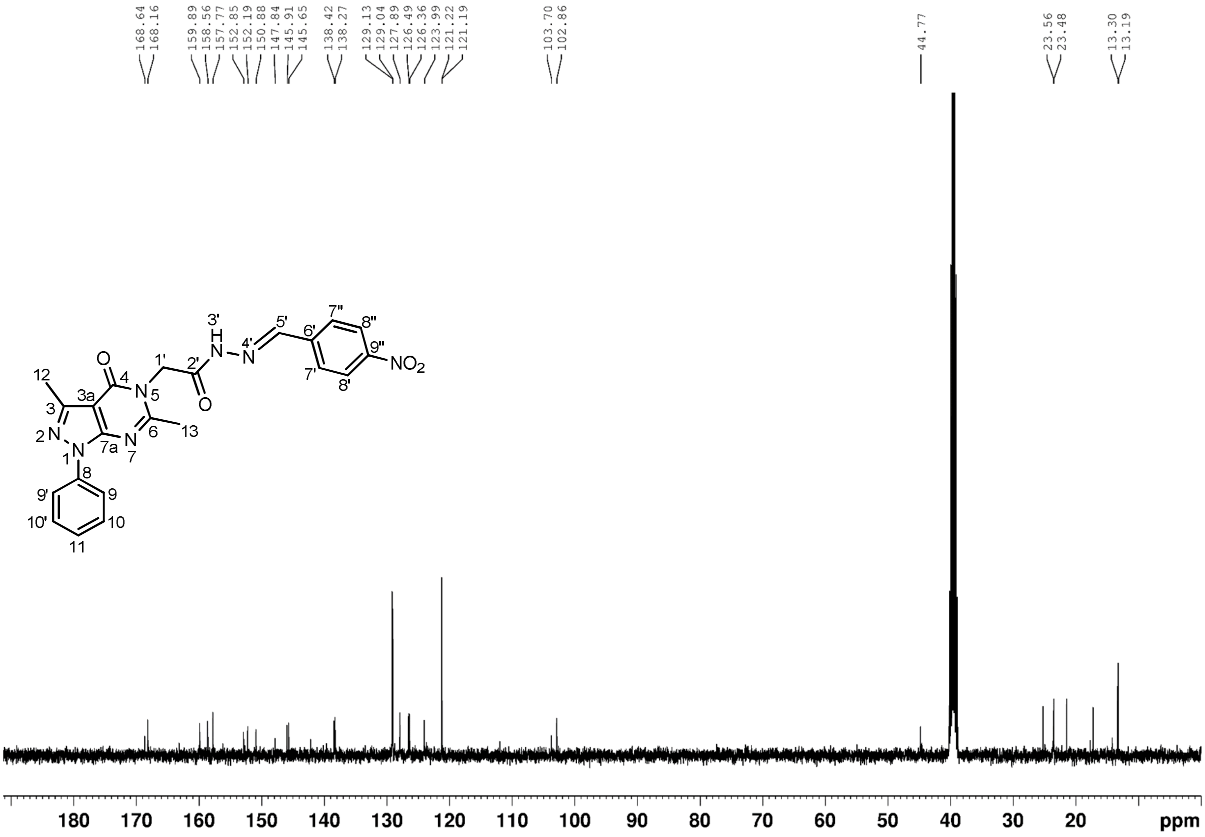

Figure S15. ^{13}C NMR spectrum of compound **5c** (DMSO-*d*$_6$, 100MHz).

International Journal of
Molecular Sciences

MDPI

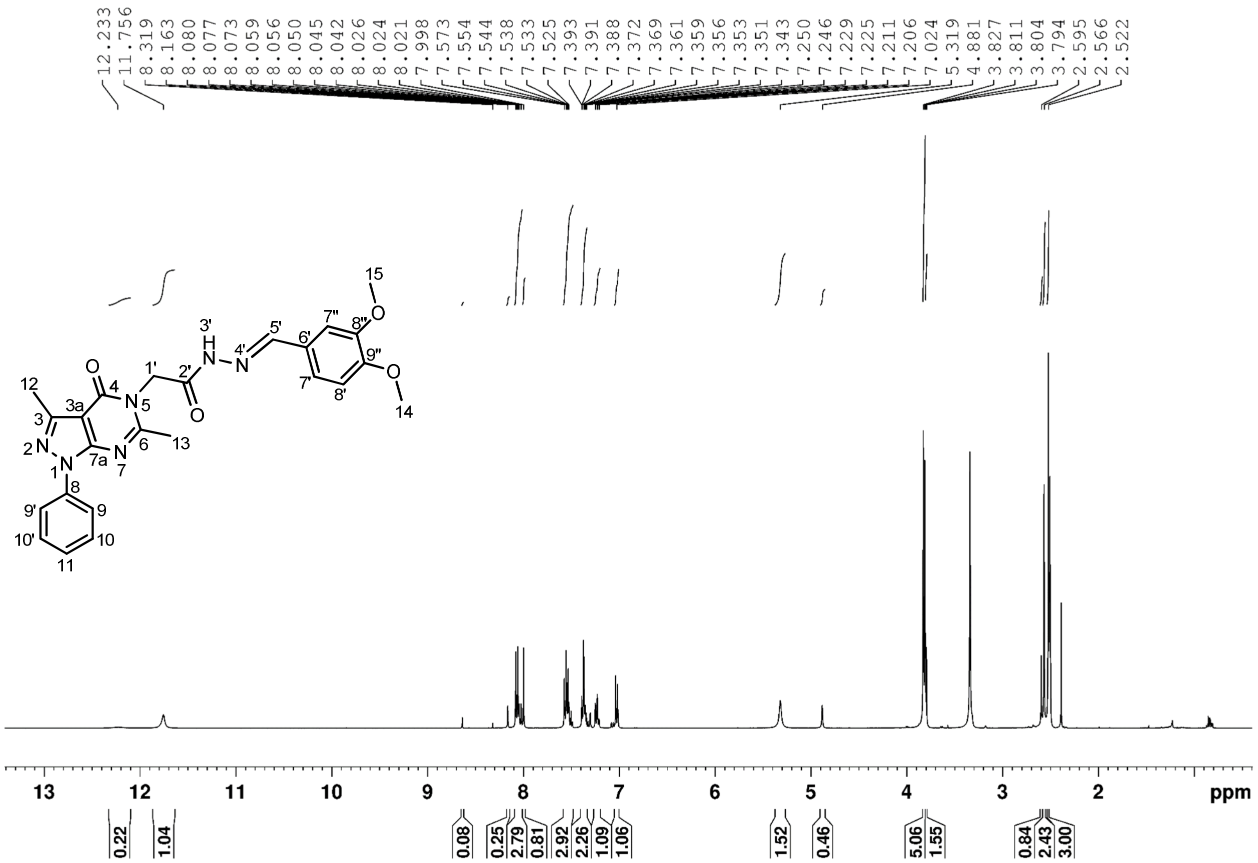

Figure S16. ^{1}H NMR spectrum of compound **5d** (DMSO-d_6, 400MHz).

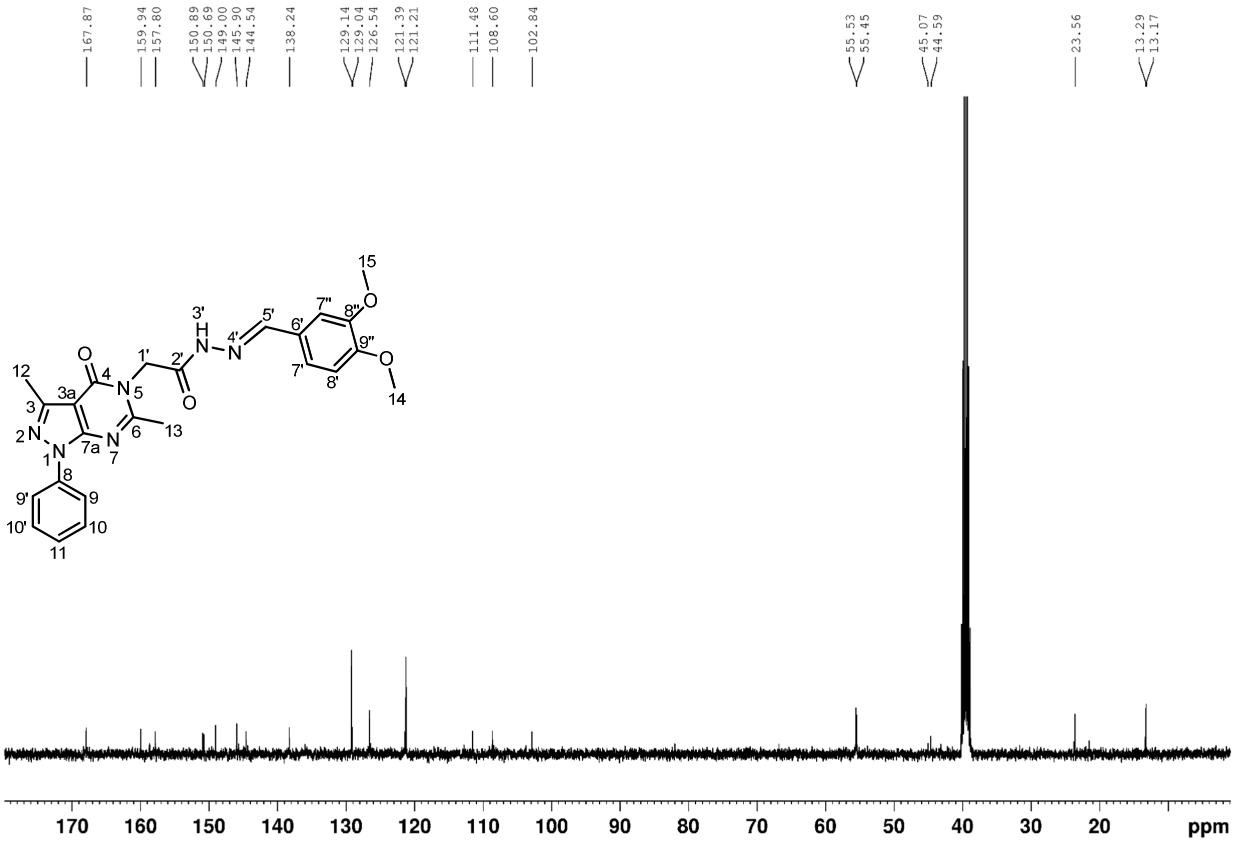

Figure S17. ^{13}C NMR spectrum of compound **5d** (DMSO-d_6, 100MHz).

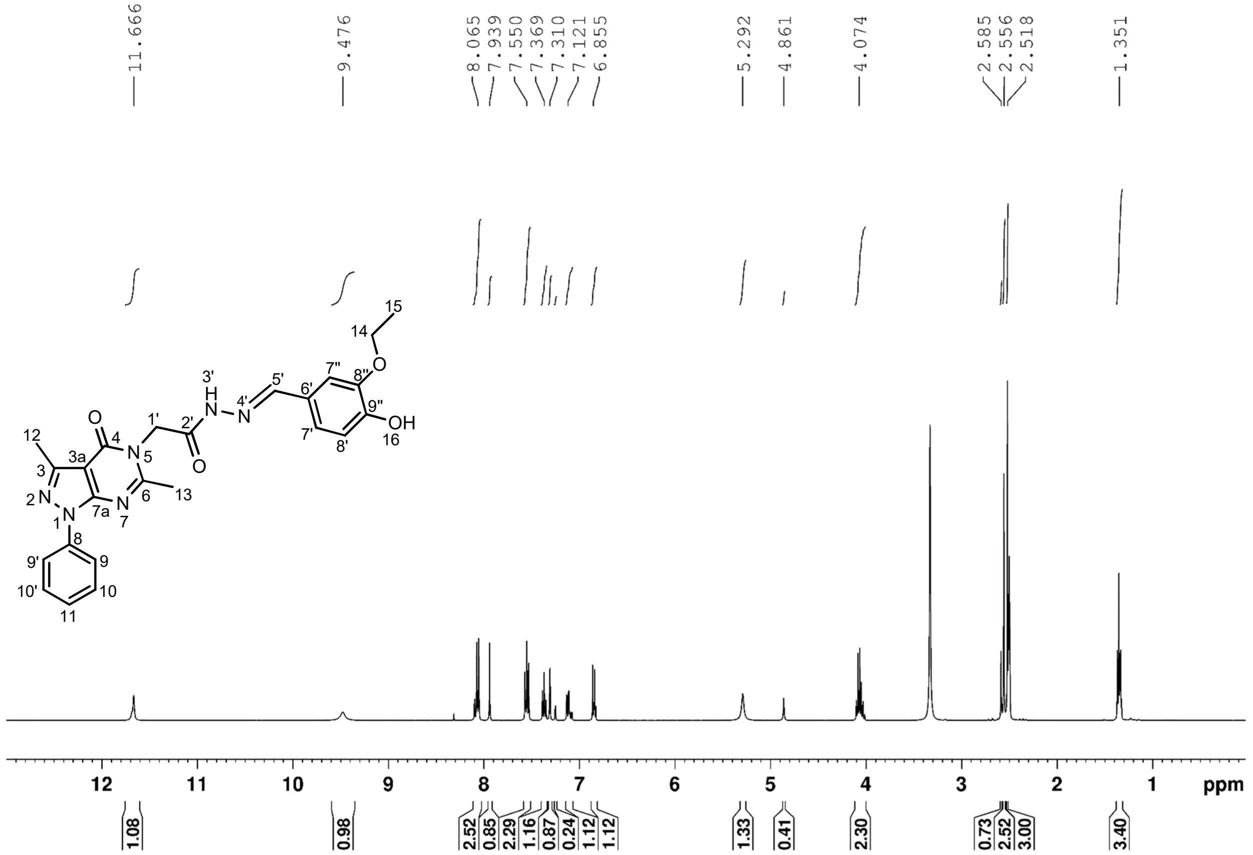

Figure S18. ^{1}H NMR spectrum of compound **5e** (DMSO-d_6, 400MHz).

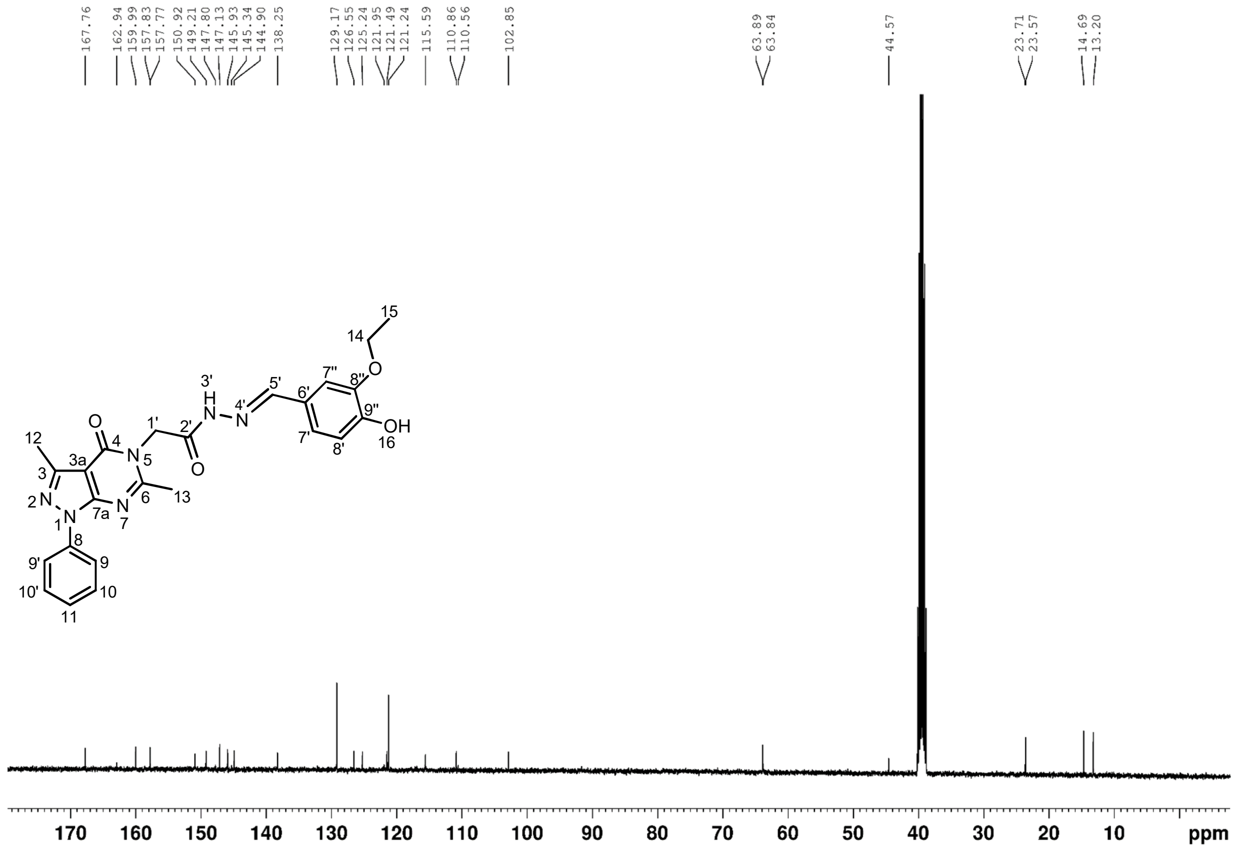

Figure S19. ^{13}C NMR spectrum of compound **5e** (DMSO-d_6, 100MHz).

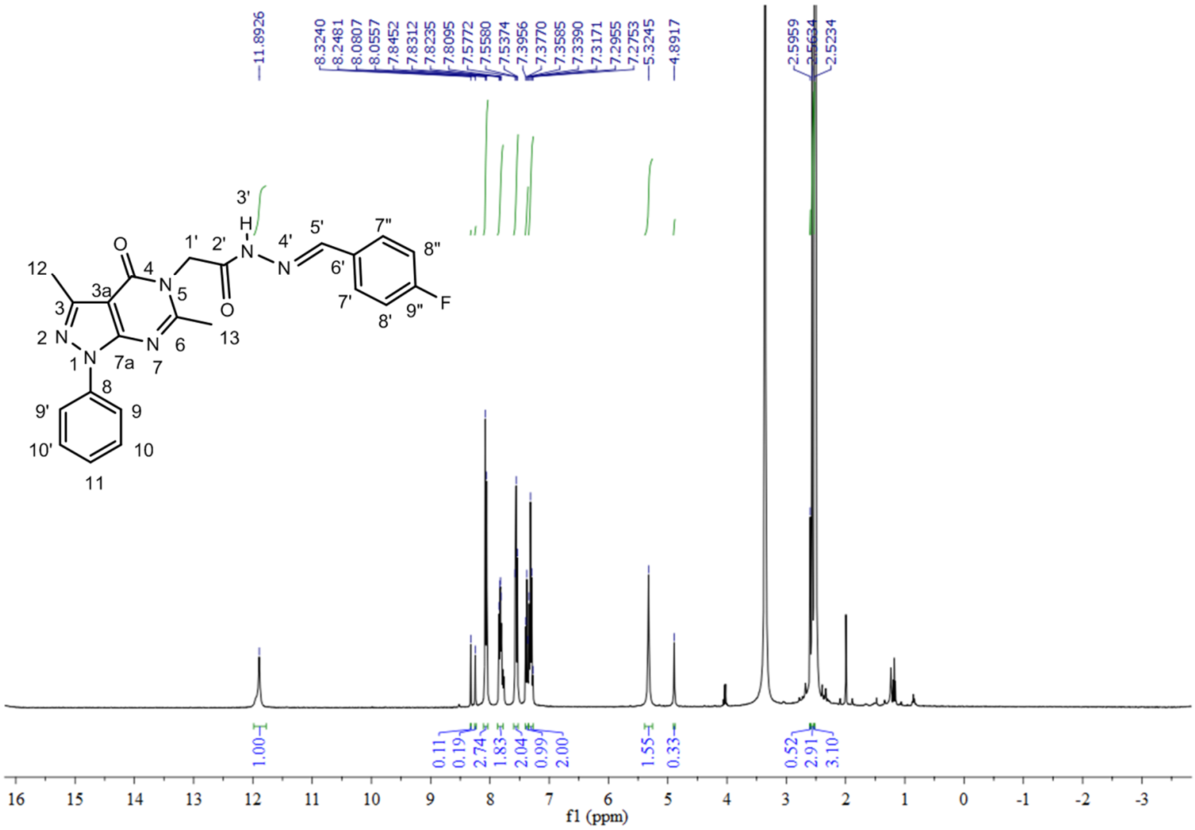

Figure S20. ^{1}H NMR spectrum of compound **5f** (DMSO-d_6, 400MHz).

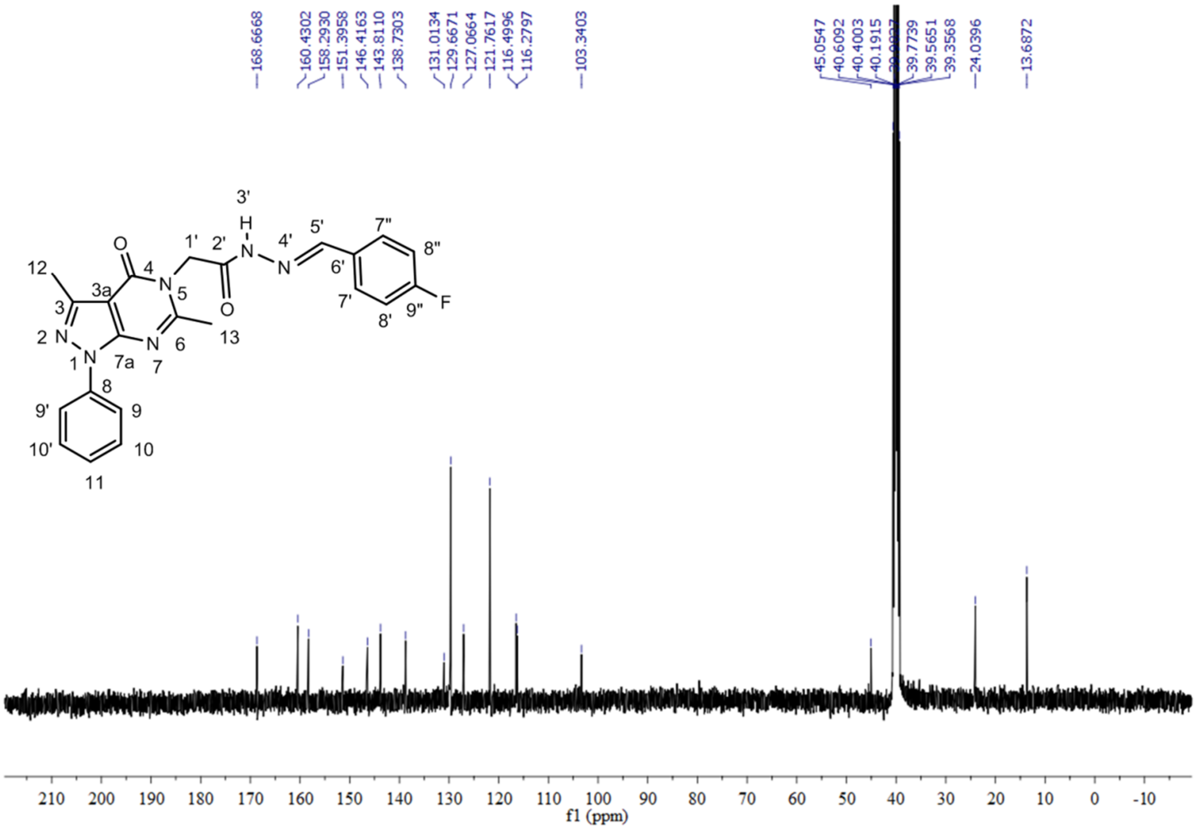

Figure S21. ^{13}C NMR spectrum of compound **5f** (DMSO-d_6, 100MHz).

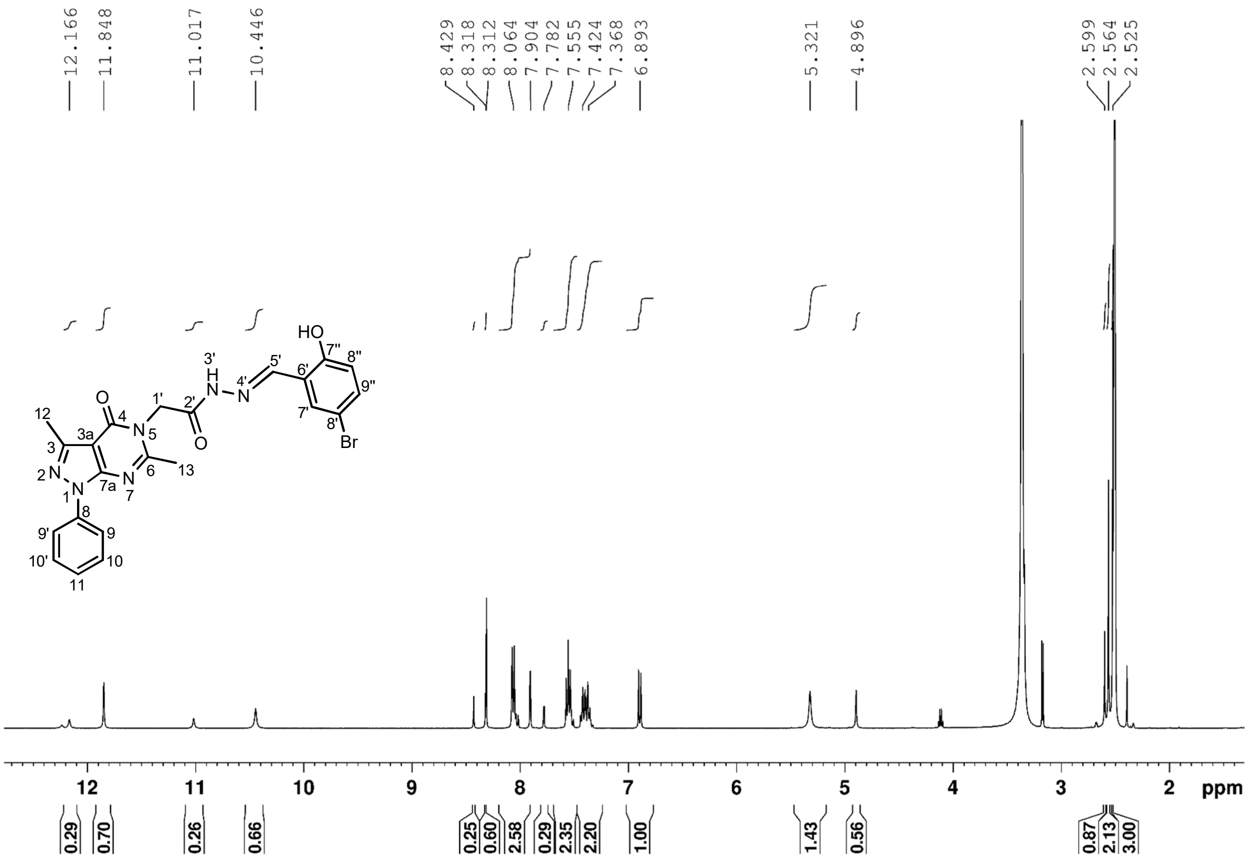

Figure S22. ^{1}H NMR spectrum of compound **5g** (DMSO-*d*$_6$, 400MHz)

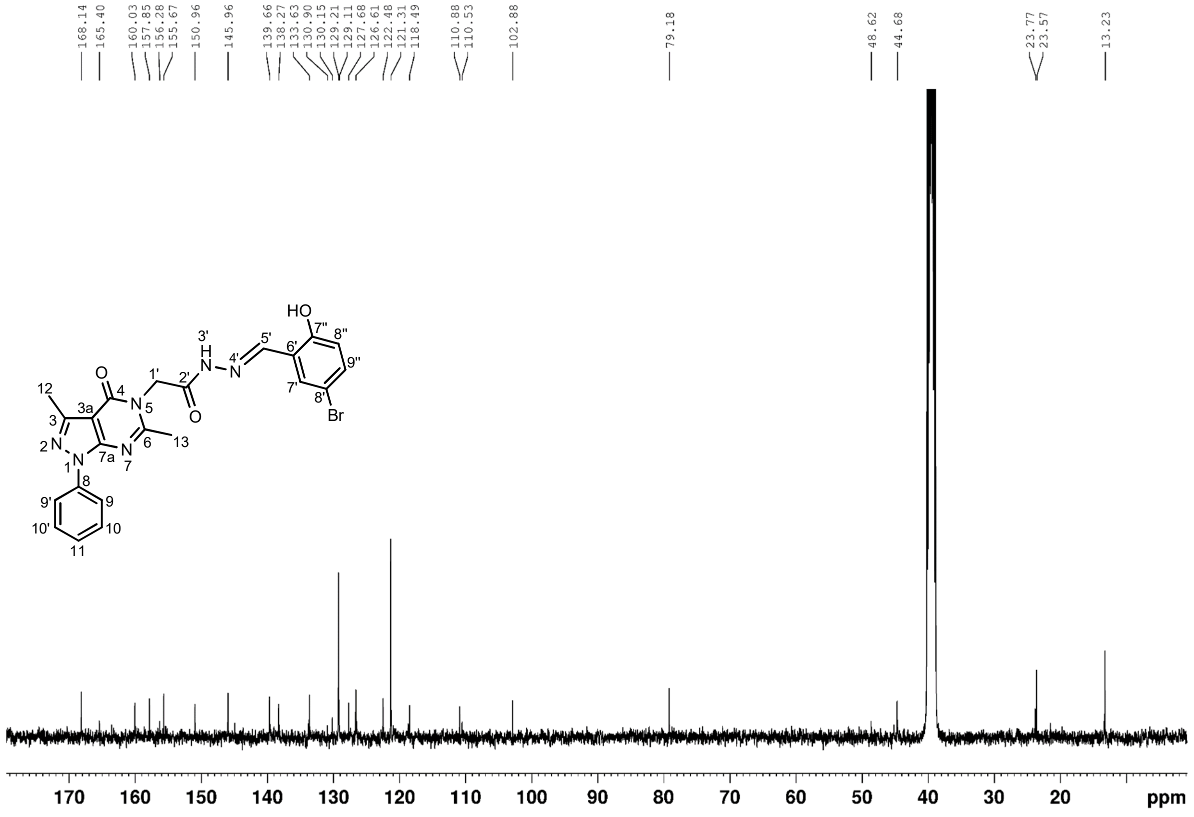

Figure S23. ^{13}C NMR spectrum of compound **5g** (DMSO-*d*$_6$, 100MHz)

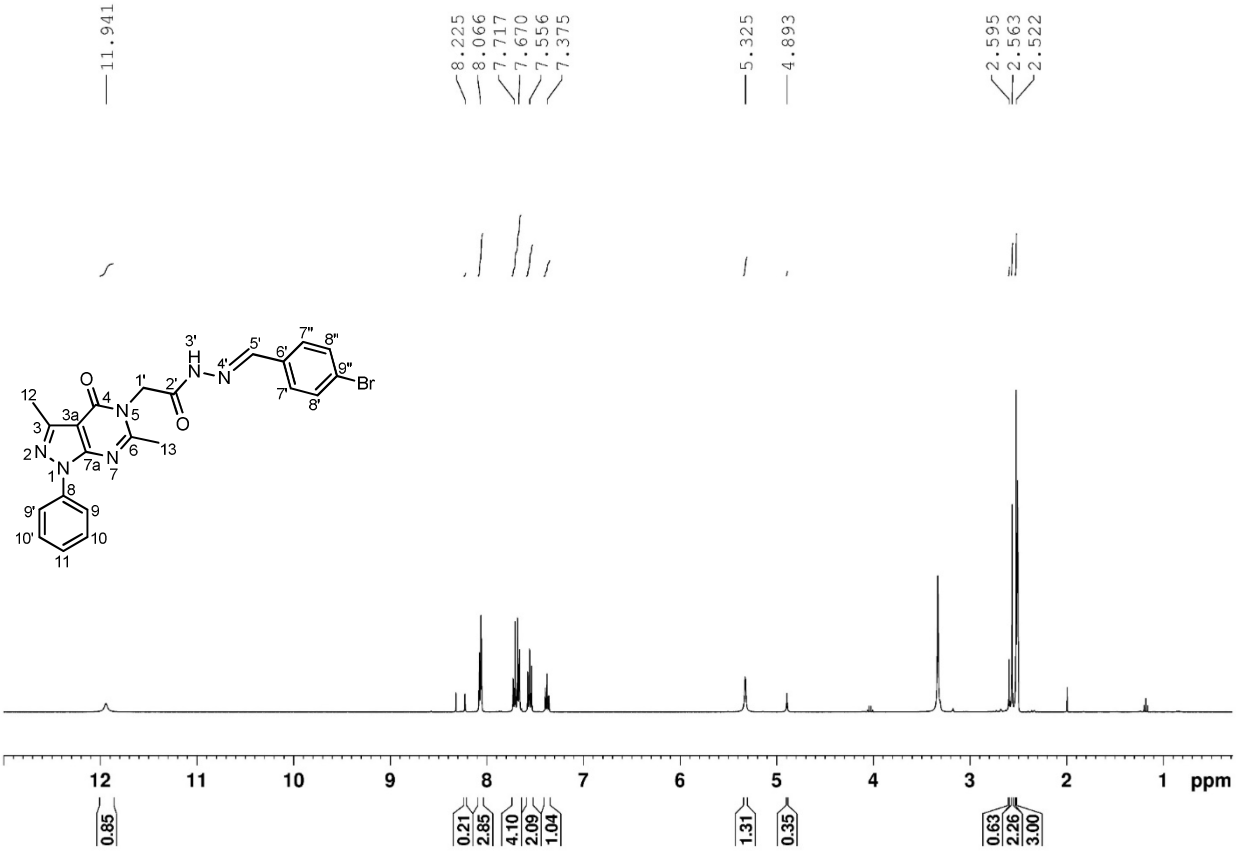

Figure S24. ^{1}H NMR spectrum of compound **5h** (DMSO-d_6, 400MHz)

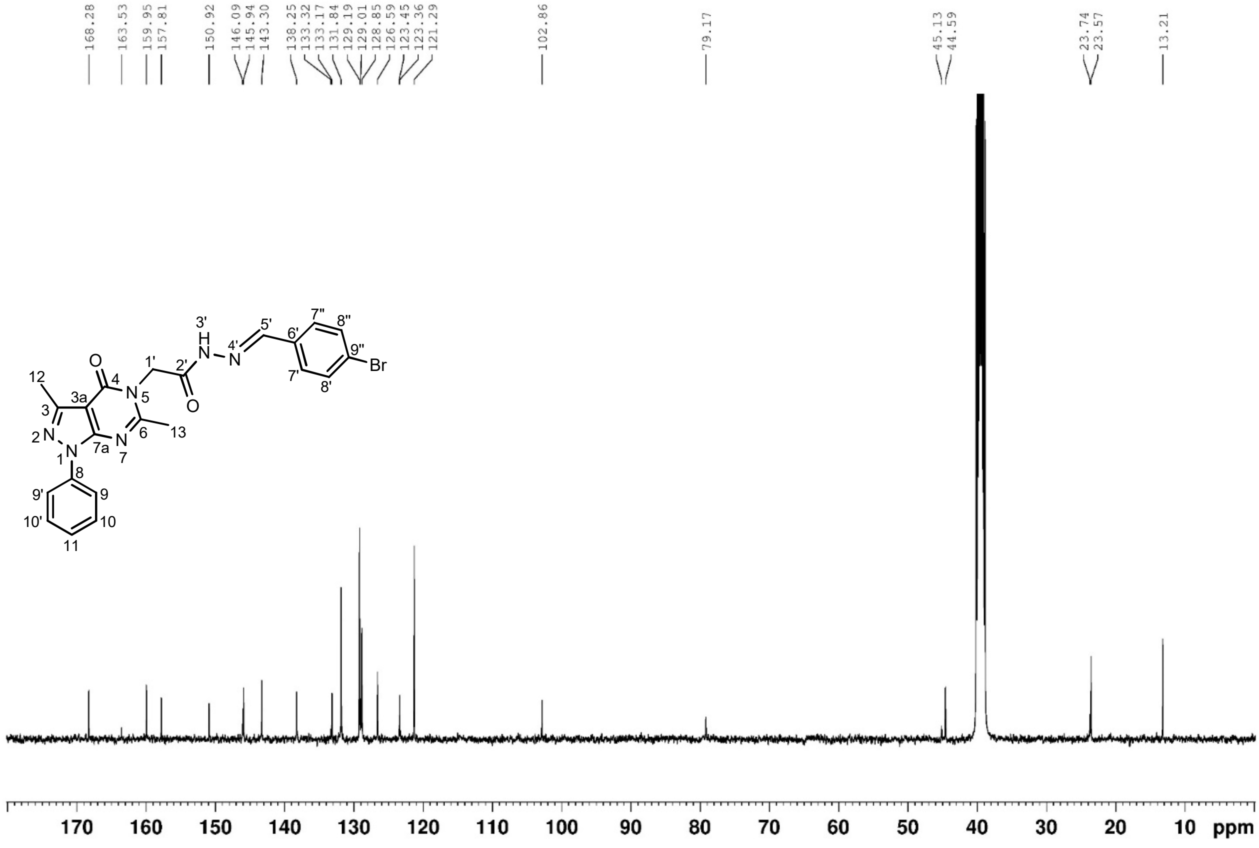

Figure S25. ^{13}C NMR spectrum of compound **5h** (DMSO-d_6, 100MHz)

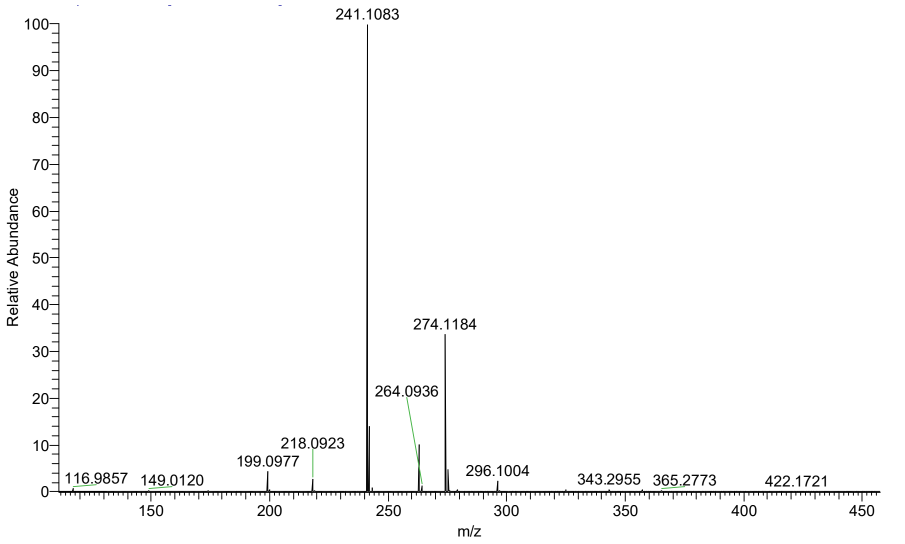

Figure S26. HR-ESI-MS spectrum of compound **2**.

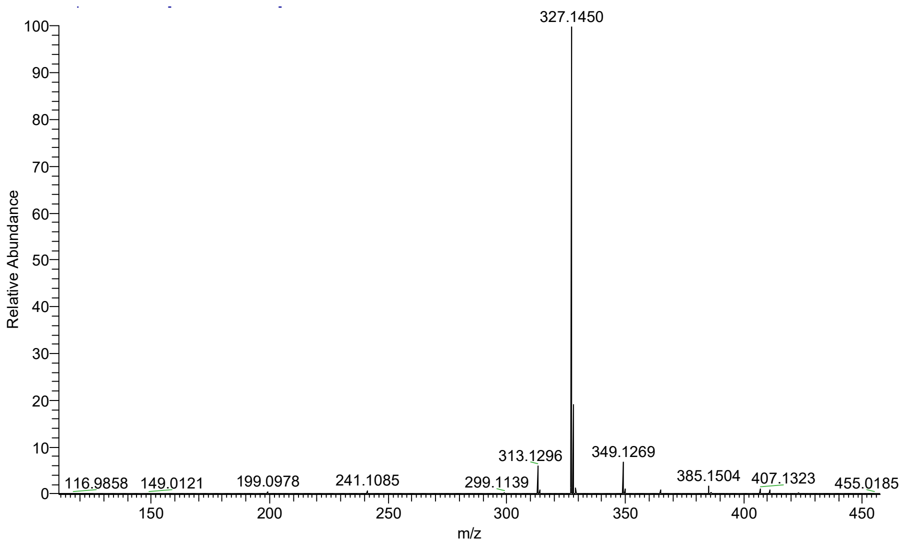

Figure S27. HR-ESI-MS spectrum of compound **3**.

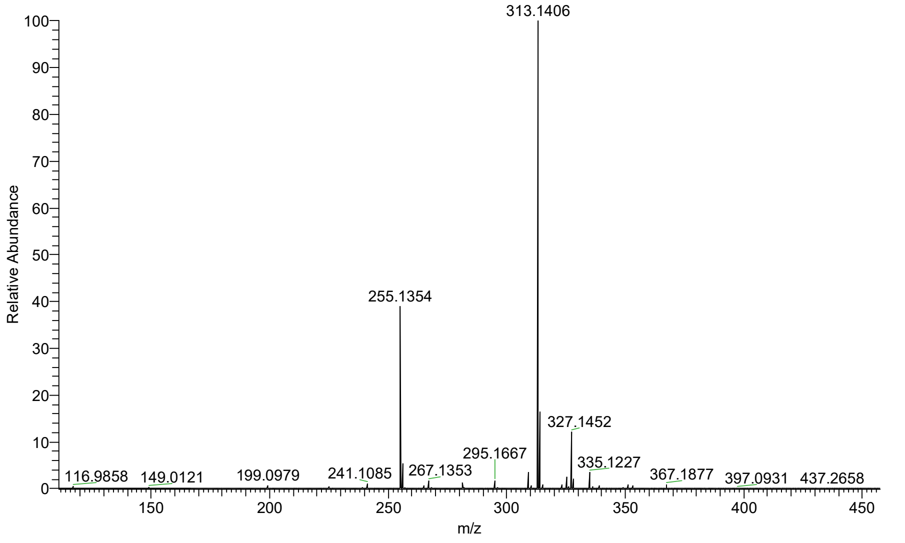

Figure S28. HR-ESI-MS spectrum of compound **4**.

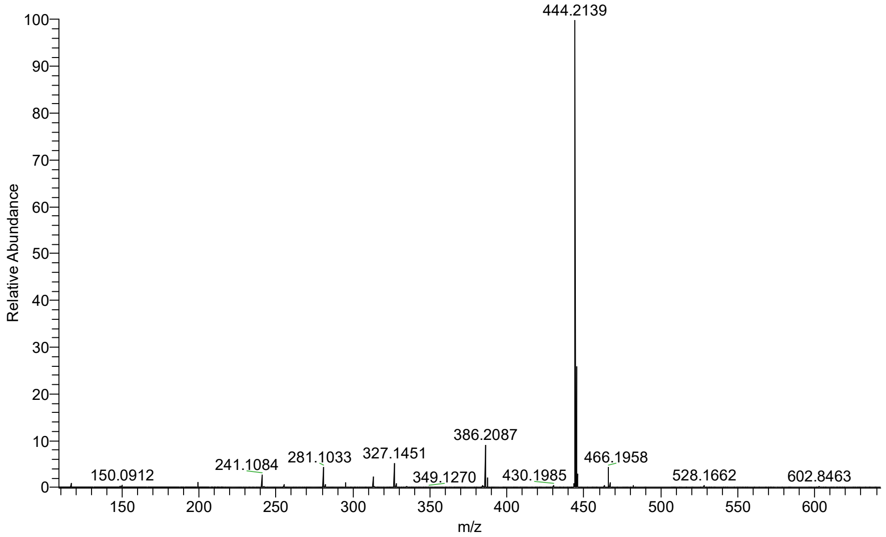

Figure S29. HR-ESI-MS spectrum of compound **5a**.

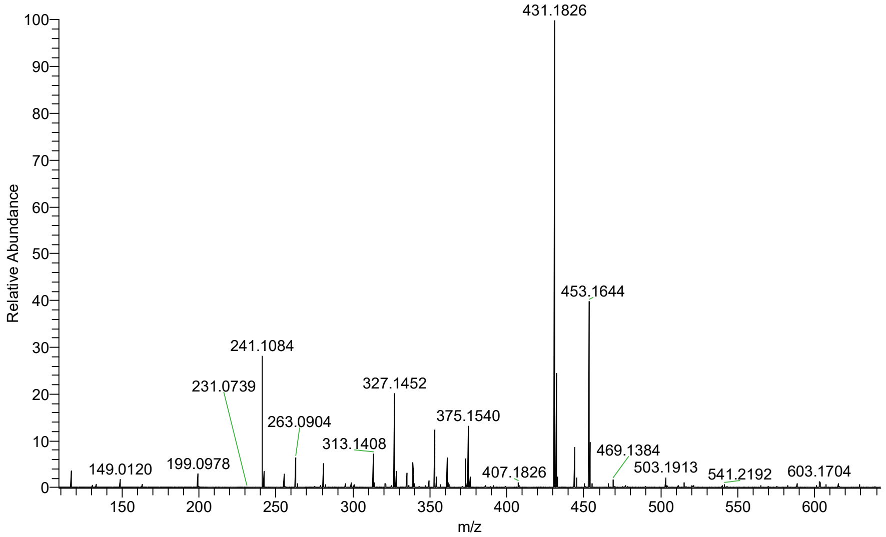

Figure S30. HR-ESI-MS spectrum of compound **5b**.

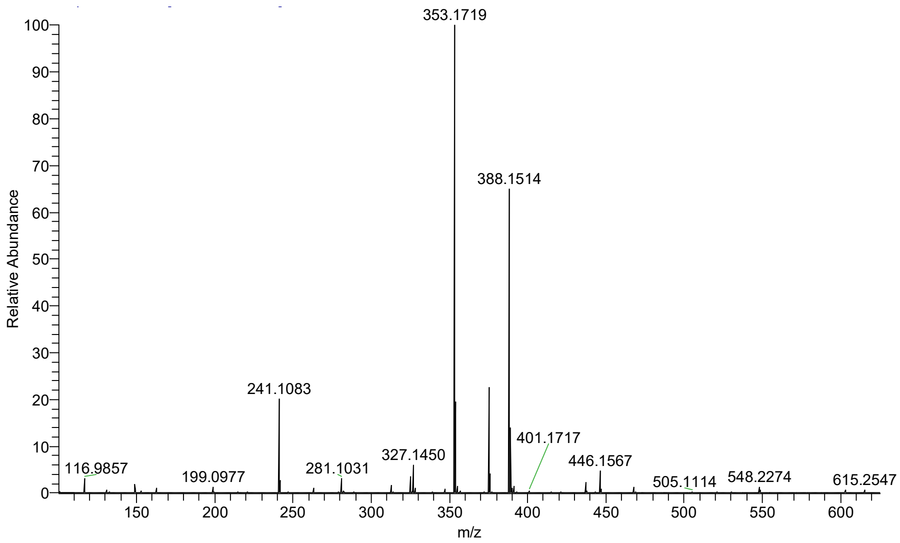

Figure S31. HR-ESI-MS spectrum of compound **5c**.

International Journal of
Molecular Sciences

MDPI

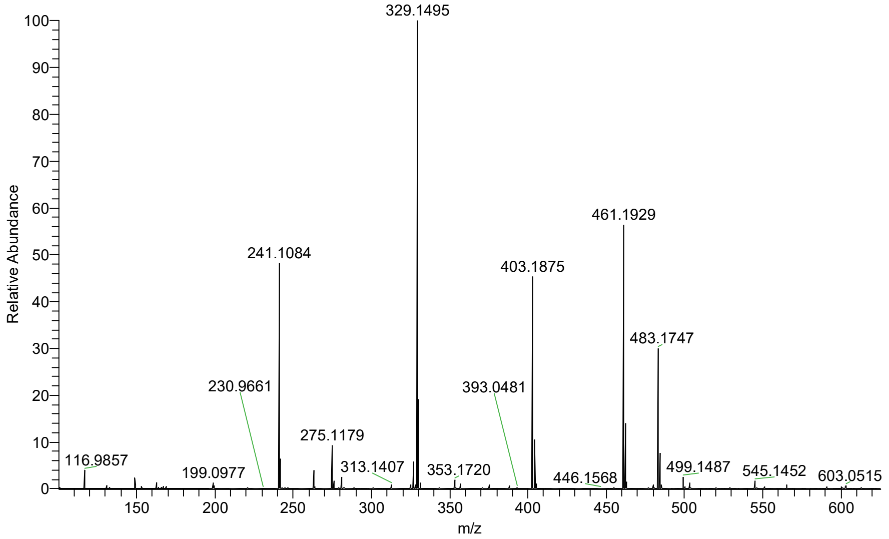

Figure S32. HR-ESI-MS spectrum of compound **5d**.

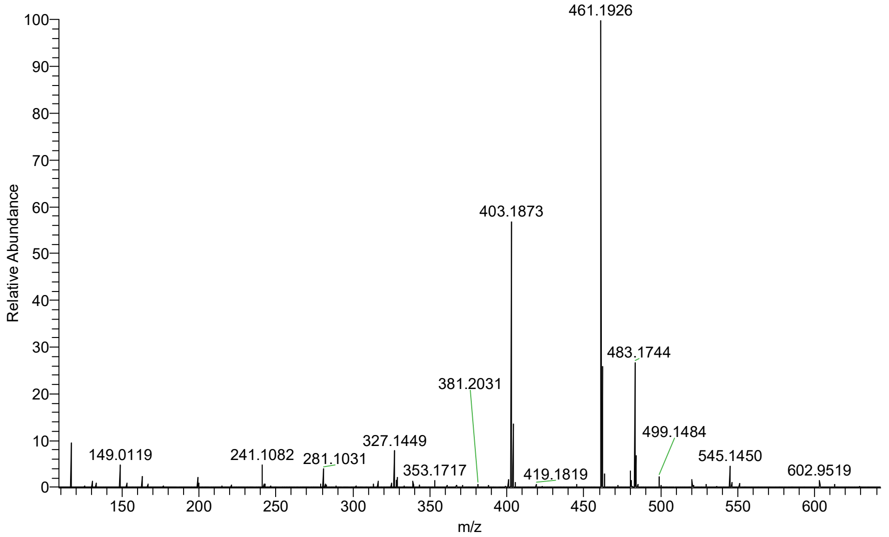

Figure S33. HR-ESI-MS spectrum of compound **5e**.

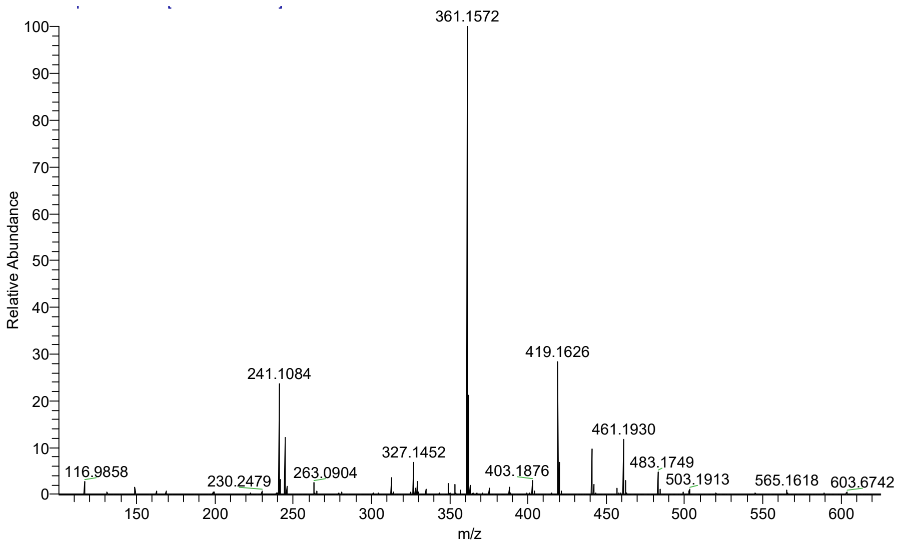

Figure S34. HR-ESI-MS spectrum of compound **5f**.

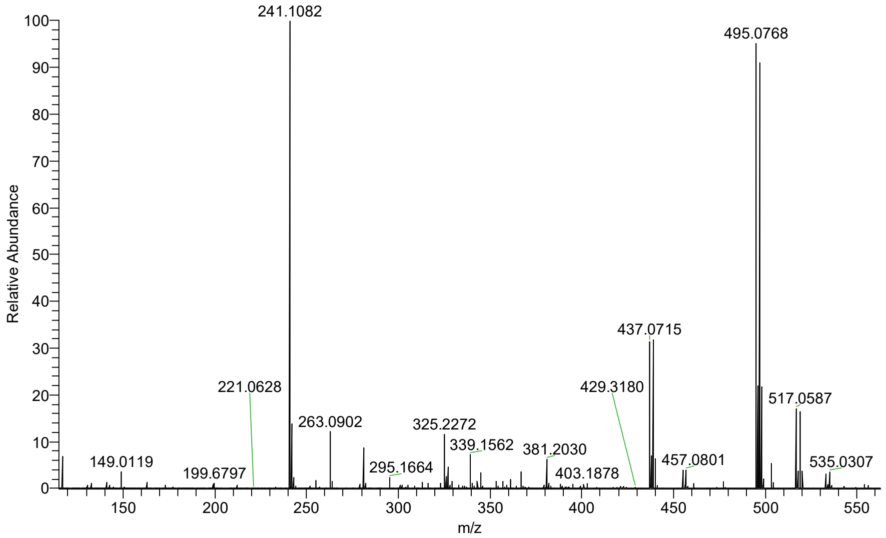

Figure S35. HR-ESI-MS spectrum of compound **5g**.

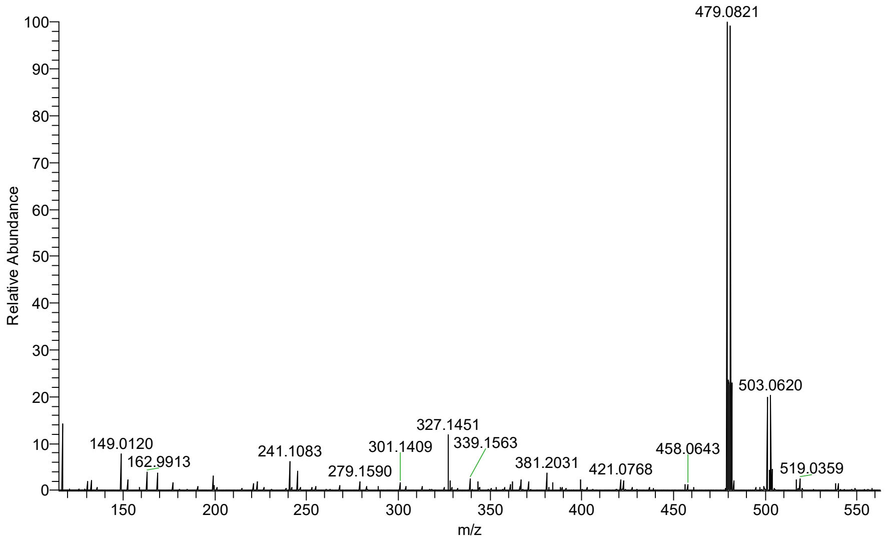

Figure S36. HR-ESI-MS spectrum of compound **5h**.
